# Feeling ‘not enough’ or ‘too much’: Exploring how LGBTQ+ adults experiencing disability navigate Canadian health contexts

**DOI:** 10.1177/13591053251327263

**Published:** 2025-03-24

**Authors:** Shannon S C Herrick, Erica V Bennett, Andrea Bundon

**Affiliations:** 1 University of Calgary, Canada; 2 University of British Columbia, Canada

**Keywords:** chronic illness, disability, gender, healthcare, intersectional, narrative method, sexuality

## Abstract

Disability and LGBTQ+ communities experience healthcare disparities, however, most research has looked at these communities separately which erases the unique health experiences of people who belong to both. This project sought to explore intersections between gender, sexuality and disability within Canadian health contexts through three life-story interviews with seven adults (aged 25–35; 21 interviews total) who identified as LGBTQ+ and experiencing disability. Thematic narrative analysis resulted in interrelated themes associated with axes of self-identification that demonstrated how participants navigated tensions between being perceived as not disabled, trans and/or queer ‘enough’ or ‘too much’ within healthcare settings. Participants relayed stories of strategically omitting and/or sharing aspects of their intersectional identities with healthcare providers to receive the care they needed. This study, in demonstrating some of the difficult compromises and decisions LGBTQ+ adults who experience disability navigate to access healthcare, highlights how ableism, cis-heterosexism and racism intertwine to shape medical systems.

## Introduction

Healthcare disparities, the preventable differences in opportunities to receive care and maintain health that are disproportionately experienced by marginalized communities, have persisted for decades across disability communities (Lezzoni and Agaronnik, 2020), lesbian, gay, bisexual, transgender, queer and non-cis-heterosexual (LGBTQ+) communities (Yerra and Yarra, 2022), as well as Black, Indigenous and People of Colour (BIPOC) communities (Macias-Konstantopoulos et al., 2023). Members of marginalized groups must make difficult decisions regarding their health so that their needs are (partially) met while navigating systems of structural inequities (Williamson, 2023).

Established health disparities across disability and LGBTQ+ communities are in part shaped by their shared histories of forced institutionalization, also known as medical incarceration. People experiencing disability have endured a long history of inappropriate and harmful seclusion, restraint and institutionalization under the guise of ‘care’ (Montenegro et al., 2023; Scull, 2011). Historically, LGBTQ+ experiences were also pathologized and viewed as something to be treated and ‘cured’ through institutionalization (McCauley and Brinkley-Rubinstein, 2017). Despite this shared history, disability and LGBTQ+ communities are commonly regarded as separate. For example, when the 1969 Canadian criminal code was reformed to allow individuals to ‘commit’ homosexuality under specific circumstances, a clause was added to specifically omit anyone experiencing mental disability (Rossi, 2022). This stark division persists as a harmful stereotype that people experiencing disability are non-sexual beings, without any sexuality, hetero or otherwise (Esmail et al., 2010).

Carrying forward these divisions, most health research has either focused on the needs of disability communities *or* LGBTQ+ communities. In addition to the structural, financial and physical access barriers that people experiencing disability face within health contexts, common misconceptions about disabilities underpin discriminatory, ableist attitudes of healthcare providers towards patients (de Vries McClintock et al., 2016; Lezzoni and Agaronnik, 2020; McClintock et al., 2018). For example, a survey of 25,006 healthcare providers’ attitudes towards disability found that most providers held implicit biases against people experiencing disabilities and had a preference towards working with non-disabled patients (VanPuymbrouck et al., 2020). Disability health research has long advocated for collaborative, people-focused care and the integration of disability-specific health curricula into medical schools (de Vries McClintock et al., 2016; McClintock et al., 2018; Ratakonda et al., 2022). Similarly, LGBTQ+ health research has demonstrated how the pervasive lack of LGBTQ+ knowledge, coupled with frequently judgemental and non-accepting attitudes of providers towards LGBTQ+ experiences, render many health contexts uncomfortable and in some cases, unsafe to access (Ayhan et al., 2019; Broholm et al., 2023; Daly and Champion, 2021).

Solely focusing on single axes of identification within healthcare research has likely generated gaps in our understanding of how to improve healthcare for people who hold multiple marginalized identities. Given that intersectional identities are dynamic and interactive, rather than strictly additive (Collins and Bilge, 2020), it is possible that several recommendations for improved health practices do not fully attend to the needs of people who experience disability *and* identify as LGBTQ+. Subsequently, this project sought to explore how people who self-identify as experiencing disability and LGBTQ+ navigate health contexts, while attending to the intricacies of participants’ intersectional identities.

## Material and methods

This project received ethics approval from the University of British Columbia’s Behavioral Research Ethics Board (BREB; approval no. H23-00670). I^1^ conducted three life-story interviews with seven adults who self-identified as experiencing disability and LGBTQ+ (21 interviews total) over the course of 3 months (Atkinson, 1998, 2007). Life-story interviews were chosen to garner an in-depth understanding of participants’ health experiences and intersectional identities over time (Atkinson, 2007). Participants were primarily recruited from digital groups created for and run by disability and LGBTQ+ communities. Following suggestions by Kidney and McDonald (2014) for accessible and respectful engagement in research, after a pre-interview session where: (a) a detailed project overview was given, (b) the consent form was reviewed, (c) participant eligibility was confirmed and (d) access needs were discussed, participants who submitted a signed consent form were then scheduled for their first interview.

All participants engaged in three interviews throughout this project. The first interview was an introductory session focused on building familiarity with questions about participants’ processes of self-identification and their formative health-related experiences (e.g. familial cultures around health). The second interview was dedicated to further exploration of participants’ conceptualizations of and experiences navigating health practices with a focus on the present. The final interview further explored health perceptions and experiences with an emphasis on the future (e.g. what participants would like to experience). Some interviews took place digitally over Zoom and others in person, depending on participants’ needs (Kidney and McDonald, 2014). Interviews were transcribed verbatim and anonymized using pseudonyms chosen by the participants. The average interview lasted 1 hour and 45 minutes, with a range of 37 minutes to 3 hours. Participants received an honorarium of $50 per interview.

### Participants

All participants experienced multiple disabilities and ranged in age from 25 to 35 years old (*M* = 28 years; SD = 3.91). The stories participants shared reflected experiences within the public Canadian healthcare system, which varies from province to province. Although four participants were born in other provinces, they relocated to British Columbia in adulthood which has a history of more progressive politics towards LGBTQ+ communities (Busby, 2020). Below, brief participant profiles are listed (in order of appearance within the results) and should be referenced to further contextualize findings:

*Jene*: 25-year-old white Canadian queer genderfluid individual with ADHD and lower-limb mobility impairments as a result of therapies and surgeries used to treat their stage 4, Ewing sarcoma in 2021.
*Forest*: 26-year-old Japanese Canadian queer woman who was diagnosed with juvenile myoclonic epilepsy coupled with anxiety and dissociation.
*Kadence*: 31-year-old white Canadian neuroqueer woman who was diagnosed with obsessive compulsive disorder in adolescence, diagnosed with general anxiety and panic disorder in adulthood, and experiences chronic pain in her hands and forearms.
*Drake*: 31-year-old Chinese Canadian pansexual genderfluid individual who experienced chronic depression and knee issues since adolescence, and experiences mobility impairments and chronic pain due to a car accident in 2019.
*Raspberry*: 35-year-old white Canadian gay xenogender* collection of beings diagnosed with dissociative identity disorder and chronic post-traumatic stress disorder, who experiences chronic depression and anxiety, as well as chronic pain and fibromyalgia (*Definition located in Supplemental Table 1: Glossary).
*Adrian*: 25-year-old Chinese Canadian pansexual bigender trans neurodivergent individual with mental illness.
*Islet*: 26-year-old Chinese Canadian queer asexual non-binary autistic individual with obstructive sleep apnoea and mental illness connected to complex trauma.

Although we believe the above profiles to best reflect the narrative methodology of this project, participant information has also been presented in Supplemental Table 2.

### Analysis

A thematic narrative analysis (Riessman, 2008) was performed to explore the patterns within participants’ stories of how they navigated health contexts. Given that personal stories were elaborated and expanded upon with each interview, the three transcripts for each individuals were combined and treated as a single text during the subsequent data analysis. I reviewed all transcripts multiple times and created detailed margin notes attending to the complexities and nuances of participants’ intersectional lived experiences within health systems. After becoming fully immersed in the data, we decided to forgo the use of software as physical copies enabled us to handle all data simultaneously which fostered the exploration of complex narratives within and across participants’ layered stories. Working with hard copies of the 21 interview transcripts encouraged us to explore intricate connections between the stories shared by all participants. This offline process of physically handling the data aligned with the narrative approach underpinning this work, as it facilitated the investigation of dynamic, coexistent realities of selves, as well as communities (Riessman, 2008). We used an intricate colour-coded scheme that symbolized different narrative threads related to participants’ intersectional identities that often tangled (e.g. purple) and pulled apart (e.g. red and blue) depending on the health context. Throughout the analysis, my co-authors acted as critical peers where alternative organizations of participants’ stories and interactions between potential themes were explored (Smith and McGannon, 2018). We discussed and explored the meaning participants assigned to their storied experiences, and the overarching societal discourses which shaped how stories were constructed. Participants were invited to review our findings via email and encouraged to offer their feedback in the form of a member reflection (Smith and McGannon, 2018). Of the four participants who engaged in member reflections, all agreed with and supported our analysis, findings and their presentation. For details on how the authors’ positionality potentially influenced the research process, see Supplemental Table 3.

## Results

**Figure 1.**
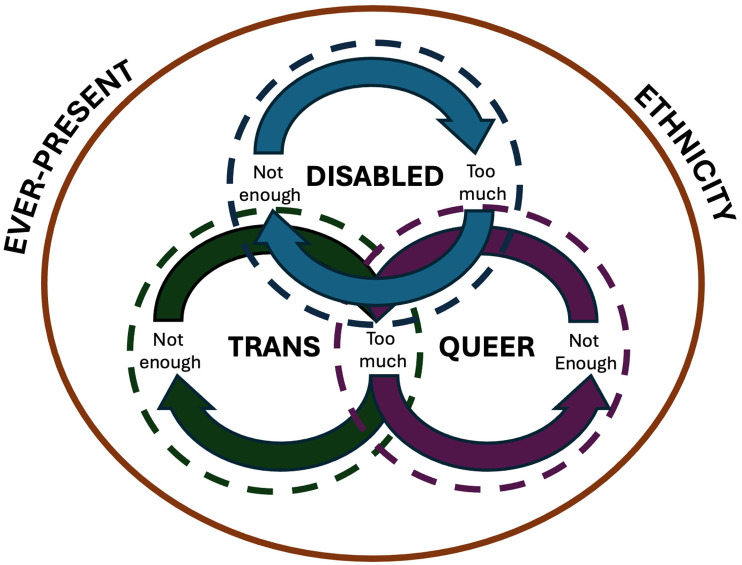
Visual representation of narrative themes.

Across participants’ stories, there appeared to be a constant tension between being perceived as ‘not enough’ or ‘too much’ regarding different aspects of their intersectional identities within healthcare contexts. Navigating these dynamic tensions, participants relayed stories of strategically sharing and/or obscuring facets of their lived experiences to receive the healthcare they needed. Across and within participants’ stories of navigating health contexts, we interpreted four interrelated themes that occurred simultaneously, but we have parsed them out along axes of self-identification to simplify their presentation: (i) Disability with two sub-themes, (a) not disabled ‘enough’–‘I don’t think the world sees me as disabled’ and (b) ‘too disabled’–‘It was just so unnecessary and felt so violent’; (ii) Trans enough or too much? –‘I don’t like being traumatized over and over’; (iii) Queer enough or too much? –‘I just haven’t felt safe in any sort of practice to be able to bring up queerness’; and (iv) Ever-present ethnicity –‘It’s an undercurrent to my entire identity’. For a visual representation of our thematic results, see Figure 1.

### Disability

#### Not disabled ‘enough’–‘I don’t think the world sees me as disabled’

Participants, despite self-identifying as experiencing disability, communicated how they often did not feel disabled ‘enough’ to consistently access resources and supports within healthcare specifically, and within society more broadly. All seven participants described their disabilities as mostly invisible, meaning they were rendered apparent through disclosure. For example, Jene explained how, ‘The disability that I have is invisible, like you can’t – I don’t need an aid all the time. I have an accessible parking pass, but I generally don’t park in those spots unless it’s the only spot or if I’m having a tired day’. A few participants acknowledged how this narrative of not feeling disabled ‘enough’ was directly tied to restrictive societal definitions and static representations of disability, as well as their own internalized ableism. Forest shared how:

There was a long time where I felt like I wasn’t deserving or worthy of being able to say that I have a disability because it was more invisible—not something that was super, outwardly physical that people could notice. And even though I have epilepsy, I don’t have like the worst possible case that it could be … but that’s one example of how health discourses have fed my internalized ableism and understanding of what disability meant.

Although participants experienced moments where they did not feel disabled ‘enough’, in adulthood most were able to decouple their identification with disability from dimensions of shame and guilt. However, some participants still experienced concern that in using disability-specific resources they were inadvertently taking up space from someone who hypothetically was more in need or ‘more disabled’ than them. Kadence shared how:

I don’t think the world sees me as disabled, so I struggle to identify as disabled, even though I know that, once again, somebody else with the exact same problems that I have will say, ‘I’m disabled’, and I’m like, ‘Yes, you are.’ But I have this idea of well … maybe I’m not disabled enough? And I really fight with that little demon in your brain, that’s like, ‘If you’re taking this identity, it means you’re taking it away from somebody who really needs it.’

Another facet of this self-described ‘imposter syndrome’ (referenced verbatim by Jene, Kadence and Raspberry) was the underlying fear of being discredited and denied their disabilities. Participants consistently felt the pressure to prove, justify and thoroughly document their experiences of disability to access the healthcare and resources they required. Drake communicated their frustrations associated with consistently proving that they were disabled ‘enough’ to health professionals and having to challenge limited understandings of what disability can be:

Not all disabilities show up in what we call medicine. Some doctors will only look at and consider what they can observe and what they see. It’s a lot of like, ‘What does your blood test show? What does your X-ray show?’ They don’t take into account anything that isn’t presented in that way. I’ve had a lot of experiences, not just with family doctors, but also with rheumatologists, and the thing I keep hearing is, ‘Keep doing physio, do the exercises.’ And I’m like, ‘Well, I am. I’ve been going to physio for the last five years.’ They don’t have anything after that aside from lose weight. That’s kind of their scapegoat, right? It’s a very two-dimensional way to look at people. They see you in person, but they don’t really even have to, and even with physical assessments, they don’t consider that you have good days and bad days. There are some things like it’s really hard to quantify your abilities in a way that doesn’t make it seem like it’s less severe than it is.

Most participants shared stories reflecting an arduous journey of finding medical professionals that would take their health concerns and disabilities seriously. This became even more complex when fatphobia intertwined with ableism and apathy to further exacerbate participants feeling disregarded, as exemplified by Drake’s story. Participants experiencing chronic conditions and pain similarly lamented the static approach (i.e. one time point for testing) within medicine that glossed over the variability and episodic nature of their experiences.

Although the feeling of being disabled ‘enough’ was rooted in participants’ complex processes of self-identification, the deeply personal and highly variable threshold of feeling ‘enough’ was often entangled with formalized medical and legal processes. For example, six participants received the Canadian disability tax credit and most found this to be affirming, while some expressed concerns about the possibility of this tax credit being denied to them one day. The fear of being discredited as disabled was directly tied to the fear of being denied the care and supports participants needed to thrive.

#### ‘Too disabled’–‘It was just so unnecessary and felt so violent’

On the opposite end of this narrative thread were experiences where participants feared being perceived or were perceived as ‘too disabled’ within health contexts. Many participants feared that if they were perceived as ‘too disabled’ then: (a) at best, they would be denied access to the services they desired, and (b) at worst, their autonomy would be stripped away through medical incarceration. Some participants, like Raspberry, personally experienced being denied health information due to their experiences with mental illness:

I saw a psychiatrist a year ago and it was to discuss what medications I could take if I decided that I wanted to try and conceive at some point. I was just curious about that. So, if I wanted to try [to conceive], I could know how far in advance I should stop taking certain medications and just do all the transitions early, if I was planning on taking that route. And it was just so horrible. She was so cruel and told me that I shouldn’t … I shouldn’t ever try to get pregnant, unless I’m able to not dissociate at all. And I was like, ‘You’re a psychiatrist and you know that I have a dissociative disorder that doesn’t go, like they don’t go away. I’m going to be dissociating a lot for the rest of my life. There’s no pill for this.’ It just felt so bad. She also said that I needed to be certain that I would never self-harm in any way ever again or that my child would learn it from me, and they would hurt themselves. It was just so unnecessary and felt so violent.

Raspberry’s story demonstrated how the medical industrial complex perpetuates ableist narratives concerning who is ‘fit’ to be a parent. Although only a few participants directly experienced being perceived as ‘too disabled’ within heath contexts, many were cognizant of that razors edge between being ‘enough’ and ‘too much’. If possible, participants strategically downplayed or completely obscured their experiences of disability to safely access reproductive healthcare. Many participants disclosed their transness and/or queerness within reproductive healthcare out of necessity, and often felt their energies were best used navigating cis-heterosexism as opposed to ableism within these contexts. For example, Kadence shared a story about switching from an intra-uterine device to an upper-arm contraceptive implant:

The gynecologist made some comment about, ‘Well, you and your man ….’ And I was like, ‘Well, my man’s actually a woman, but that woman still does have a penis and testicles.’ And she’s like, ‘Oh, okay, but you need to understand, gynecology is very heteronormative.’ [laughs] Yeah, no shit.

Trans and gender-nonconforming (TGNC) participants struggled against being perceived as ‘too disabled’ to be granted access to gender-affirming healthcare. Although there has been movement towards the de-pathologization of TGNC identities in recent years, the associations between and conflation of TGNC experiences with mental illness still had to be navigated by participants. For example, Adrian shared how:

This was back when I didn’t really know how to advocate for myself, but I’ve discussed being trans with a psychologist and they basically said, ‘How do you know it’s not part of your illness or something?’ So that’s another intersection, when you have mental illness, a lot of people will downplay your gender identity or even your sexual orientation. Like I’ve had psychosis, and I haven’t disclosed this to my doctors, but if someone had disclosed [that] they have psychosis and they’re also transgender, a lot of the times, the doctors will assume it’s part of some delusion or hallucination. That’s another crossover that people don’t realize, is when you’re mentally ill and you’re trans or even just gay or anything like that, it’s a strike against you.

To access gender-affirming healthcare, Adrian has learned to omit certain aspects of their mental illness (i.e. psychosis) when discussing their trans experiences. Similarly, due to their previous experiences with psychiatry, Raspberry opted to disengage from that field entirely to reduce the likelihood of experiencing medical gaslighting*:

I loathe the mental health field more than the other health fields, and I will not even go see a psychiatrist anymore, because every experience I have with them, they’re just so cruel and so untrusting. And do not make me feel like I could have any of the right information about what I’m experiencing.

For participants experiencing mental illness, the tangible threat of medical incarceration seemed to underpin the bulk of their healthcare experiences. Islet shared how, ‘You say the wrong thing and you go to hospital prison, like that’s so bad’. When talking about what they would ideally like healthcare to look and feel like in the future, Islet explained how:

I think non-carceral [healthcare] would be great. I’d love to have that—forms of community care and accountability and conflict resolution. Because I feel like a lot of people are rightfully concerned about obtaining care because medical systems are set up where some people are disproportionately way more likely to be harmed or killed or gaslighted. And nothing that is done on top of the existing system can solve it, essentially because it’s part of the root of it, of how it was built up. I’d love to live in the world where people were only scared of the doctor ‘cause they didn’t like needles [laughs].

As mentioned by Islet, despite the efforts of the Canadian de-institutionalization movements, the carceral roots embedded within our healthcare systems are difficult to weed out entirely. When asked about their ideal future of healthcare, all participants, regardless of their differing experiences of disability, expressed a strong desire for anti-oppressive and de-carceral practices to become common place. For example, Forest shared how:

Just general anti-oppressive care and understanding of how healthcare experiences might be different for queer folks or BIPOC folks or disabled folks. I don’t often find that healthcare settings are very friendly for a lot of disabled folks, as you probably heard from many people [laughs]. So being mindful of how healthcare settings are experienced by the people that they’re intended to be for.

### Trans ‘enough’ or ‘too much’–‘I don’t like being traumatized over and over’

As mentioned above, participants that identified as TGNC had even more complex relationships with health contexts as they often had to strategically choose between accessing gender-affirming healthcare *or* receiving adequate support for their disabilities. Self-advocacy was paramount for TGNC participants to prove that they were trans ‘enough’ to be granted access to the healthcare they needed. Islet explained how, ‘For me and a lot of other people, advocating for yourself is the only way to access trans care. You have to speak for yourself and speak on your experiences to someone who may or may not be receptive’. Islet went on to explain how:

If you want to get a legal gender marker changed, you need to have a doctor sign off on it and say, ‘Yup, this isn’t for fraud.’ That’s pretty much all it is. Gotta prove that it isn’t for fraud [laughs] which I get, but also there’s something to be said about like I shouldn’t need a doctor to say that.

In parallel to concerns about being perceived as ‘too disabled’ to received gender-affirming care, some TNGC participants hid their trans experiences within certain health contexts to receive appropriate medical care. Raspberry (RB) shared in the following discussion with me (1A) how they felt like a ciswoman in medical contexts, struggled with dysphoria, and strategically omitted their xenogender experiences for fear that they were too far beyond society’s narrow understanding of trans experiences:

1A: Have your experiences with health contexts ever influenced how you self-identify?

RB: I mean, they’ve given me gender dysphoria before. And I definitely feel more like a ciswoman when I’m in appointments. I have a nurse practitioner who’s pretty good with remembering my pronouns, but still, there’s so much gendered language in health and mental health stuff. So, yeah, I get pretty big dysphoria and go into this old, feeling like a woman space, a lot of the time when I’m accessing healthcare. So, it influences it in that sense, just temporarily causing me imposter syndrome and gender dysphoria.

1A: Does it sometimes feel, and this is totally offside, that with the doctor your gender has to be confined in a way to make you legible as a ciswoman so that you can access resources that you need?

RB: Yes! Yeah, absolutely, and like I don’t have xenogender written down anywhere with my nurse practitioner or bring it up. Like I just gotta do what I gotta do here, but I can do it as non-binary but like that’s as far as I can go. She [nurse practitioner] knows I have DID and different parts will wake up in front of her and we’re good but, yeah, maybe in the future that would shift. I’m just so new to trusting a healthcare person, period.

The delicate balancing act between feeling trans ‘enough’ and being perceived as ‘too trans’ within health contexts was complicated, and at times, taxing for participants to navigate. Similarly, Adrian shared how for them it was not worth the additional energy fighting for their trans experiences to be recognized:

Certain identities will have a strike against me in the medical system. Sometimes I absolutely have to mask who I am. But there’s some parts I can’t mask, like I can’t mask that I’m Asian, but if I go into the doctor and they want to see me as a cis person … it sounds bad, but I don’t like going through the trouble. I don’t like being traumatized over and over. Sometimes I just have to go with what the system wants.

Unlike participants’ disabilities, genders and sexualities, their ethnicities could not be strategically omitted within health contexts (see theme ‘Ever-present ethnicity’ for details).

### Queer ‘enough’ or ‘too much’–‘I just haven’t felt safe in any sort of practice to be able to bring up queerness’

Participants, particularly in their teenage and young adult years, grappled with feeling queer ‘enough’ to belong to LGBTQ+ communities and access LGBTQ+ specific resources and supports. In adulthood, for many participants feeling queer ‘enough’ was directly tied with community connection and self-acceptance. Some participants, like Drake, still struggled against restrictive representations of what queerness should look like:

I have never been one of the queer people who are like, ‘Let’s go to the Pride Parade. We’re queer, we’re here,’ you know? I’ve never been super, crazy enthusiastic about that. I’ve always felt never quite queer enough. So, I don’t really interact with a whole lot of the queer community.

Although all participants noted how LGBTQ+ communities and resources were one potential avenue for social support, most also critiqued the predominantly white able-bodied homonormative spaces for perpetuating racism and ableism.

In addition to seeking reproductive healthcare, participants’ queerness was also implicated when they attempted to support their partners, and vice versa, within health contexts. For example, when Jene was undergoing extensive chemotherapy treatments, they were grateful to the nurses who allowed their partner to visit them. Now in remission, Jene explained how they communicate their relationship within healthcare settings, as well as some of the heterosexist assumptions they had previously encountered:

Everything you navigate when you’re queer is coming out to people because you have to, you’re kind of made to in certain ways. Quite frequently Carol [pseudonym], my girlfriend, would be asked if she was my sister, and we look nothing alike. I’m like, ‘No, does that look like my sister?’ [laughs] We actually talked a lot about language, between the two of us, she’s my girlfriend, I’m her partner, just for gender reasons, right? But in healthcare, government, and professional settings, she’s also my partner, because if I call her my girlfriend, people think I mean friend and she doesn’t want that, and I don’t want that. She has to kind of change her identity a little bit to communicate what our relationship is.

By referring to each other as partners within healthcare, Jene has found that this slight shift reinforces the nature and seriousness of their relationship. Among participants that were not in relationships, queer experiences were more commonly omitted from healthcare contexts to avoid potentially uncomfortable exchanges. Many, like Forest, did not feel safe enough within healthcare contexts to speak about their queer experiences:

In terms of queerness, I feel like I just haven’t felt safe in any sort of practice to be able to bring up queerness or mental health really with my family doctor, or, not that it really comes up with neurologists or specialists, but I haven’t seen the kind of intentionality from my healthcare practitioners that would make me feel safe to talk about those issues in those spaces.

All participants acknowledged how queer experiences were becoming steadily more accepted within society, but several still felt that this acceptance had yet to be fully integrated into healthcare contexts.

### Ever-present ethnicity –‘It’s an undercurrent to my entire identity’

As much as participants navigated tensions between upper and lower limits of perceived acceptability associated with aspects of their intersectional identities, their ethnicity was not subject to negotiation. Most participants identified as East Asian and their experiences reflected certain ethnically charged power dynamics within healthcare. For example, Adrian explained some of the stereotyping they have experienced:

Sometimes they [medical professionals] have expectations for us, like when they see Asian people, a lot of times they feel like Asian people will not campaign for changes in their healthcare, like we won’t really fight back or something. So, I feel sometimes people can mistreat us. So, that’s another issue when you are Chinese, and then especially with mental illness … it’s just a whole can of worms.

Harmful stereotypes, such as the presumed docility of Asian people that Adrian faced, further complicated and lessened the quality of care they were able to receive. Participants often struggled against dominant white narratives within healthcare while simultaneously navigating cultural and familial health narratives. Forest also shared how:

Dealing with mental well-being in the context of intergenerational trauma, and for me, that’s looked like mental health being more of a taboo topic in my family. You are just told to hide it away. I’ve learned through conversations with my family elders that a lot of that was in response to the internment and having to have that attitude of ‘We have to move on. We can’t focus on mental health when we’re just trying to get our physical needs met.’ And there’s been a lot of unlearning through my family system as we’re trying to stop that pattern of shutting out the mental well-being side of things.

All participants spoke to some degree about the taboo quality of discussing their mental health within their families, however the complexities of navigating intergenerational trauma within health contexts were only expressed by East Asian participants.

Contrastingly, white participants acknowledged how their ethnicity afforded them privileges within healthcare, specifically and Western society, more generally. For example, Jene explained how:

Being white and a settler, it’s not something I necessarily identify with, but I realize that it’s an undercurrent to my entire identity, like I can’t parse out, just put aside being white. It’s the only really truly visible majority identity. I recognize that shapes my experience in every other part of my identity, as a white queer person, as a white disabled person. My experiences are very different from many BIPOC persons’ experiences with that, in general, but especially in healthcare.

## Discussion

Across participants’ stories of navigating Canadian healthcare contexts as LGBTQ+ adults who experience disabilities, there appeared to be constant tensions between being perceived as not disabled, trans and/or queer ‘enough’ and ‘too much’. The complex negotiation of intersectional identities by participants was predominantly used to facilitate access to the healthcare they needed while attempting to avoid or minimize harm.

All participants self-identified as experiencing invisible disabilities. Without visible markers of disability, individuals often experience societal stigma and discrimination due to their appearance (i.e. presumably non-disabled) being in contrast with their lived reality (Javaid and Yusuf, 2024). However, the siloed nature of Western medicine could be used to participants’ advantage, where tactical manoeuvring facilitated the care they needed. Although effective, this deliberate concealment of disabilities has been long associated with greater emotional stress, feelings of shame and lower self-esteem (Ysasi et al., 2018). Due in part to the invisibility as well as variability of participants’ disabilities, many grappled with feelings of not being disabled ‘enough’ to access disability-specific resources and supports.

These sentiments echo the theorizing of disability justice scholar, J. Logan Smilges. In their book *Crip Negativity* (2023), they problematize the neoliberal scarcity that governs the idea that claiming a disability identity inherently takes something away from others, who might have a greater need. Within medical systems, neoliberal scarcity discourses have been found to drive focuses on efficiencies and the cost-effectiveness of healthcare that further marginalizes people who regularly depend on these services (Denier, 2008). Framed by scarcity as opposed to abundance, our participants experienced concerns over whether they were disabled ‘enough’ as well as consistent pressures to thoroughly document and prove their experiences of disability to access resources, supports and care. Similarly, findings from a phenomenological study that interviewed 14 adults who self-identified as experiencing invisible disabilities, highlighted how participants were often verbally called out and questioned by strangers for needing accommodations like designated seating on public transportation (Kattari et al., 2018). Given our project’s focus on health experiences, the process of being questioned on one’s disabilities by medical professionals, who are gatekeepers to care, was considered inherently stressful and, at times, frustrating.

Participants expressed a mutual lack of trust wherein they could not fully trust their medical professionals and felt that their healthcare providers did not trust them in return. A qualitative exploration of dignity from the perspectives of Australian adults experiencing disability (*N* = 19) found that a lack of acknowledgement of lived experiences negatively affected participants’ well-being and identified healthcare settings as particularly challenging environments for maintaining dignity (Chapman et al., 2024). Acknowledgement of personhood was found to be critical for dignified experiences which often resulted in feelings of respect, safety and independence (Chapman et al., 2024). Putting this work in conversation with our own, LGBTQ+ adults who experience disability seem to anticipate that their whole personhood will not be acknowledged within health contexts and proactively fragment their intersectional identities to minimize potential harm.

Many participants acknowledged how the carceral history of Western medicine’s approach to mental health underpinned their healthcare experiences and manifested as a fear of being perceived as ‘too disabled’. Within British Columbia, psychiatric deinstitutionalization without sufficient mental health and community resources, has increased the precarity of people experiencing mental illness to become unhoused and incarcerated (Read, 2009), which in turn, have mass disabling effects. Given the ongoing nature of deinstitutionalization, participants were wary of bringing their whole selves into health contexts for fear that aspects of their intersectional identities would be used as grounds to either deny them care or incarcerate them. Participant concerns about being perceived as ‘too disabled’ were also likely amplified due to the recent history of LGBTQ+ experiences being pathologized and criminalized (Cossman, 2024; McGuire, 2022).

When seeking gender-affirming and/or reproductive healthcare, where LGBTQ+ experiences were difficult to withhold, participants were especially aware of the potential to be perceived as ‘too disabled’ to receive care, and strategically obscured their disabilities when possible. For TGNC participants, dependency upon shifting trans rights discourses and medical systems to gain access to resources and services placed them in perilous positions, where access was often contingent on proving ones’ transnormativity*, which works to re-impose binary gender while simultaneously excising disability to better incorporate transness into a neoliberal nation-state (Puar, 2017). Research exploring experiences of gender-affirming readiness assessments in Aotearoa, New Zealand with transgender young adults (*N* = 13) also found that the pressure to adhere to transnormativity to access gender-affirming care was prevalent (Fraser et al., 2021). Similarly, a study exploring how gender-nonconforming young adults (*N* = 10) experienced gender-affirming healthcare found that participants had to ‘borrow’ a binary trans label to receive appropriate care (Lykens et al., 2018). In our project, the need to express some aspects of transnormativity to access health services often forced TGNC participants to obscure facets of their trans experiences, as well as their experiences of disability.

When seeking care for their disabilities and support for their general health, participants often did not disclosure their sexuality and/or gender to healthcare providers to minimize the likelihood of discrimination. These findings echo those of previous work exploring the healthcare experiences of LGBTQ+ young adults (Rossman et al., 2017) and older adults (Burton et al., 2019) where participants avoided disclosing LGBTQ+ identities to healthcare providers to improve quality of care. Within our project, through previous experiences of being misgendered as women in health contexts, Adrian, Drake, Jene and Raspberry learned that completely obfuscating their trans experiences had the potential to minimize harm and the improve quality of care received for their disabilities. However, the likelihood of women’s pain, especially BIPOC women’s pain, to be disregarded within medical systems has been thoroughly documented (see Hossain, 2021), which likely undermines this supposed improvement of care. Research investigating patient experiences at an LGBTQ+ health centre has demonstrated how LGBTQ+ identity disclosure to health care providers can be instrumental to facilitating appropriate care (Broholm et al., 2023). Within most health contexts, healthcare providers are presumed not to be knowledgeable of, let alone comfortable with LGBTQ+ experiences, which deters patients’ from disclosing their LGBTQ+ identities unless it is unavoidable (Broholm et al., 2023; Lykens et al., 2018; Rossman et al., 2017).

In recent years, scholars have explored the tenuous identity memberships LGBTQ+ individuals negotiate and navigate with regard to feeling queer and trans ‘enough’ (Sutherland, 2023; Thöni et al., 2024). For racialized sexual and gender minorities, questions of being queer and trans ‘enough’ may become increasingly complicated by feelings of disconnect from their ethnic communities because of their LGBTQ+ identities as well as disconnection from mainstream LGBTQ+ communities due the predominant whiteness of these networks (Ghabrial, 2017; Logie and Rwigema, 2021). A qualitative study exploring the healthcare experiences of transgender people of colour through a combination of interviews (*N* = 22) and focus groups (*n* = 17) found that most participants felt they would receive better care if they were white and/or cisgender (Howard et al., 2019). Within our project, East Asian participants spoke about the difficulties associated with navigating intensified power dynamics and ethnic stereotyping when working with white healthcare providers. However, it is important to note that none of our participants identified as Black, Brown and/or Indigenous. Given the complex histories of medical experimentation (e.g. Washington, 2006), forced sterilization (e.g. Pegoraro, 2015) and disproportionate incarceration (e.g. Erevelles, 2014), future health research should attend to the intersectional lived experiences of Black, Brown and Indigenous Canadians who identify as LGBTQ+ and experience disability.

Within health contexts, navigating constant tensions between being perceived as not disabled, trans and/or queer ‘enough’ and ‘too much’, became an integral skill required for participants to access care while maintaining their autonomy and protecting their well-being. However, the complex and constant (re)negotiation of participants’ intersectional identities was mentally and emotionally taxing. Depending on the care sought, participants founds that they could show up as either LGBTQ+*or* experiencing disability but rarely, if ever, both. Due to the nature of participants’ disabilities, they had the choice to conceal this aspect of their lived experiences. Future work should explore how LGBTQ+ adults who experience visible disabilities navigate health contexts to expand our understanding of how ableism and cis-heterosexism shape healthcare. Although participants expressed an underlying desire to engage in health contexts as their whole selves without obscuring any aspects of their intersectional identities, they also made it clear that current Canadian healthcare systems were not set up to facilitate their genuine inclusion.

### Conclusions

Despite significant health scholarship that has focused on how to improve healthcare for disability *or* LGBTQ+ communities, it is possible that suggestions for improved disability healthcare might perpetuate the presumed cis-heterosexuality of patients *and* that suggestions for LGBTQ+ healthcare may reinforce ableism within medical systems. This project, in demonstrating some of the difficult compromises and decisions LGBTQ+ adults who experience disability navigate to access healthcare, highlights how ableism, cis-heterosexism and racism intertwine to shape medical systems. Participants’ stories encapsulate the limitations of inclusion based on single axes of identification instead of nuanced understandings of complex intersectional lived experiences. Participants advocated for the integration of intersectional, anti-oppressive, non-carceral and trauma-informed practices within and across health contexts. Although previous research has called for more education in healthcare on disability, gender and sexuality (e.g. Ayhan et al., 2019; de Vries McClintock et al., 2016), our project suggests that medical systems are not currently structured to support the genuine, holistic inclusion of people who are disabled, trans *and* queer. Understanding and challenging how underpinning systems of oppression manifest within healthcare is long, arduous and necessary work, so that in the future it’s never a question of ‘not enough’*or*‘too much’, of either LGBTQ+*or* disability, to safely access care.

## Supplemental Material

sj-docx-1-hpq-10.1177_13591053251327263 – Supplemental material for Feeling ‘not enough’ or ‘too much’: Exploring how LGBTQ+ adults experiencing disability navigate Canadian health contexts Supplemental material, sj-docx-1-hpq-10.1177_13591053251327263 for Feeling ‘not enough’ or ‘too much’: Exploring how LGBTQ+ adults experiencing disability navigate Canadian health contexts by Shannon S C Herrick, Erica V Bennett and Andrea Bundon in Journal of Health Psychology

sj-docx-2-hpq-10.1177_13591053251327263 – Supplemental material for Feeling ‘not enough’ or ‘too much’: Exploring how LGBTQ+ adults experiencing disability navigate Canadian health contexts Supplemental material, sj-docx-2-hpq-10.1177_13591053251327263 for Feeling ‘not enough’ or ‘too much’: Exploring how LGBTQ+ adults experiencing disability navigate Canadian health contexts by Shannon S C Herrick, Erica V Bennett and Andrea Bundon in Journal of Health Psychology

sj-docx-3-hpq-10.1177_13591053251327263 – Supplemental material for Feeling ‘not enough’ or ‘too much’: Exploring how LGBTQ+ adults experiencing disability navigate Canadian health contexts Supplemental material, sj-docx-3-hpq-10.1177_13591053251327263 for Feeling ‘not enough’ or ‘too much’: Exploring how LGBTQ+ adults experiencing disability navigate Canadian health contexts by Shannon S C Herrick, Erica V Bennett and Andrea Bundon in Journal of Health Psychology

## Notes

1. Whenever I or my is used, it is in reference to the first author. Whenever we is used, it is in reference to all authors.
