# Supplementary material for: Feeling ‘not enough’ or ‘too much’: Exploring how LGBTQ+ adults experiencing disability navigate Canadian health contexts
Source: J Health Psychol. 2025 Mar 24;30(13):4075–90. doi: 10.1177/13591053251327263 (PMC12618724; doi:10.1177/13591053251327263)
Supplement: sj-docx-1-hpq-10.1177_13591053251327263 – Supplemental material for Feeling ‘not enough’ or ‘too much’: Exploring how LGBTQ+ adults experiencing disability navigate Canadian health contexts [file sj-docx-1-hpq-10.1177_13591053251327263.docx]

Supplemental Table 1. Glossary of acronyms and uncommon terms (in order of appearance within manuscript).

| **Acronyms** | |
| --- | --- |
| **LGBTQ+** | Lesbian, Gay, Bisexual, Transgender, Queer, and all sexual and gender identities that are not cisgender and/or heterosexual |
| **BIPOC** | Black, Indigenous and People of Colour |
| **TNGC** | Transgender and Gender-Non-Conforming |
| **Terms** | |
| **Xenogender** | Non-binary gender identity and umbrella term for genders cannot be described and categorized by human understandings of gender |
| **Gaslighting** | When someone is manipulated using psychological methods into questioning their own powers of reasoning and sanity |
| **Transnormativity** | A set of standards used by both cis and trans people to evaluate the authenticity of a person’s transness that relies heavily on a binary gender logic that assumes all transgender people wish to be perceived as a cis |
