# Supplementary material for: Feeling ‘not enough’ or ‘too much’: Exploring how LGBTQ+ adults experiencing disability navigate Canadian health contexts
Source: J Health Psychol. 2025 Mar 24;30(13):4075–90. doi: 10.1177/13591053251327263 (PMC12618724; doi:10.1177/13591053251327263)
Supplement: sj-docx-2-hpq-10.1177_13591053251327263 – Supplemental material for Feeling ‘not enough’ or ‘too much’: Exploring how LGBTQ+ adults experiencing disability navigate Canadian health contexts [file sj-docx-2-hpq-10.1177_13591053251327263.docx]

Supplemental Table 2. Authors’ positionality and potential influence on research process.

|  | **Self-disclosed Positionality** | **Potential Influence on Research** |
| --- | --- | --- |
| **First Author** | Identifies as a white chronically ill LGBTQ+ scholar and was inspired to pursue this community-driven research through their own experiences navigating the Canadian health contexts post-pandemic | -Increased the likelihood of mutual understanding and comparable previous experiences between the participants and first author  -As a white person, author was cognizant of their racial privilege and how their whiteness shaped their previous experiences within health contexts  -Within all interviews, author approached this work from a place of deep compassion, empathy, and a dedication to developing understanding  -Within the interviews and analysis, great care was taken to understand the participants’ identities across the multitude of health stories shared  -Research and analysis also encouraged the first author to grapple with their own self-identification processes as well as the simultaneously distinct yet interconnected chronically ill and disability communities |
| **Second Author** | Identifies as a white, cisgender, queer, able-bodied, middle-class 39-year-old woman whose research attends to LGTBQ+ experiences of embodiment and health across the life course. | -Personal experiences with queerness and work in queer communities means some shared understanding with research participants  -Background working a social support group facilitator for LGTBQ+ adults with chronic conditions shaped her interest in better understanding the intersection of LGTBQ+ and disability identities with the aim of improving health care delivery and outcomes  -Collective personal, community, and research-related experiences shaped the suggestion and undertaking of a narrative approach to analysis to understand the storied aspects of meaning-making to center participant voices and complexities in experience  -As a 39-year-old white woman, age and race relations were unpacked throughout analysis and writing process, paying attention to how generational differences and white privilege may have led to assumptions about the data  -Navigation of the healthcare system as an able-bodied person may have led to lack of nuance in understanding of ableism within health care contexts. Assumptions were scrutinized throughout critical friend discussions with the first and third author |
| **Third Author** | Identifies as a white, cisgender, heterosexual, non-disabled woman whose research attends to (dis)ability in sport, exercise and health contexts | -Longtime engagement with disability communities and disabled scholars was aware of the ‘overlap’ between disability, chronically ill and queer communities.  -Community engagement and past research has explored and critiqued the medicalization of disability and the use of sport and physical activity more general to ‘rehabilitate’ disabled bodies.  -Sport and other ‘contexts’ where the author more commonly engages are often sex and gender segregated (i.e., men’s sport or women’s sport) or segregated by impairment or diagnosis (i.e., group exercise for individuals with MS or sport program for wheelchair uses). This project provided opportunity to explore the intersections of identities that often go unexpressed or unacknowledged in these other spaces |
