# Supplementary material for: Feeling ‘not enough’ or ‘too much’: Exploring how LGBTQ+ adults experiencing disability navigate Canadian health contexts
Source: J Health Psychol. 2025 Mar 24;30(13):4075–90. doi: 10.1177/13591053251327263 (PMC12618724; doi:10.1177/13591053251327263)
Supplement: sj-docx-3-hpq-10.1177_13591053251327263 – Supplemental material for Feeling ‘not enough’ or ‘too much’: Exploring how LGBTQ+ adults experiencing disability navigate Canadian health contexts [file sj-docx-3-hpq-10.1177_13591053251327263.docx]

Supplemental Table 3. Participant information.

| **Pseudonym** | **Age** | **Ethnicity** | **Gender** | **Sexuality** | **Disabilities** |
| --- | --- | --- | --- | --- | --- |
| Jene | 25 | White Canadian | Genderfluid | Queer | -Neurodivergent (ADHD)  -Diagnosed with Stage 4, Ewing Sarcoma in 2021 (tumour found in hip bone)  -Mobility impairments due to cancer-related treatment and surgeries |
| Forest | 26 | Japanese Canadian | Woman | Queer | -Juvenile myoclonic epilepsy (diagnosed at 13 years old) coupled with anxiety and disassociation |
| Kadence | 31 | White Canadian | Woman | Neuroqueer | -Obsessive compulsive disorder (OCD) diagnosed at age 8  -Diagnosed in adulthood with general anxiety and panic disorder  -Self-diagnosed as autistic  -Chronic pain in hands and forearms |
| Drake | 31 | Chinese Canadian | Genderfluid | Pansexual | -On-going knee issues since childhood (started at age 8/9)  -Mobility impairments and chronic pain due to a car accident in 2019  -Chronic depression (since childhood), stress, anxiety, and undiagnosed but suspected ADHD |
| Raspberry | 35 | White Canadian | Xenogender | Gay | -Dissociative Identity Disorder (DID)  -Chronic Post-Traumatic Stress Disorder (CPTSD)  -Obsessive Compulsive Disorder (OCD)  -Panic disorder  -Chronic depression and anxiety  -Fibromyalgia and chronic pain |
| Adrian | 25 | Chinese Canadian | Bigender | Pansexual | -Neurodivergent with mental illness  -Identifies with Mad Pride |
| Islet | 26 | Chinese Canadian | Non-binary | Queer asexual | -Neurodivergent (autism)  -Obstructive sleep apnea  -Exercise induced asthma  -Mental illness connected to complex trauma |
